# Institutional Notes and News

**Published:** 1911-04-01

**Authors:** 


### Institutional Notes and News.

**Chesterfield Hospital.**

As Mr. C. E. Barnes, J.P., remarked : The extra forty surgical beds at the Chesterfield and North Derbyshire Hospital, provided last year, have been fully justified. Though the beds were not opened until March, 1910, the daily average number occupied for that year had risen to 92. This unexpected increase was largely responsible for the additional rise in the year's expenditure, which resulted in a deficit of £1,359. As the charitable public has contributed liberally to the extension fund, it is hoped that the working men will do their best to provide funds for its maintenance. It needs quite £7,000 per annum to keep all the beds opened : a sum which for a neighbourhood like that of Chesterfield should not be difficult to obtain. The extra beds have not been occupied by an increased number of accidents—the hospital has more accommodation for women and children, more provision for ophthalmic patients, and very largely in these directions the increase has been due.

**New Scheme at Mount Vernon Hospital.**

An important statement by the chairman, concerning the proposed detailed scheme for reorganisation, gave particular interest to last week's annual meeting of the Mount Vernon Hospital for the Diseases of the Chest, over which Mr. J. S. Fletcher, M.P., presided, in the absence of Mr. Henry Stedall. It will be remembered that the hospital has establishments at Hampstead and Northbrook. The report, said Mr. Fletcher, called attention to the work done in the heart wards. Many people were not aware that the hospital treated heart complaints and bronchitis as well as consumption. New mechanical appliances had enabled better work to be done in heart cases. In fact, patients who knew they had heart trouble often lived longer than others, because it caused them to live a more careful life. The hospital was about to consider in detail a scheme of re-organisation which the Medical Board had been thinking over for some time, and had recommended to the Board of Management, which latter Board were disposed to consider favourably. The chief obstacle was the fact that it would cost money. The Medical Board wished to pay more attention to curable patients. The first wish was to take in everybody who applied, but their sad experience had been that consumption, when once it had got hold of an individual, was hardly ever cured. The scheme of this hospital was to keep in touch with curable patients, and give advice as to clothing, diet, mode of life, and suitable occupation; in fact, to go out among the people concerned in the hope of arresting disease. It was intended that there should be a better classification of beds, setting aside certain beds for heart complaints, but the majority kept for pulmonary disease, a number of which would be set apart for treatment by tuberculin. The scheme also embraced graduated labour proportioned to the strength of the patient, at Northwood. It was very important also in diverting the attention of the patient from his own complaint to his task. Another feature was that there should be a dispensary for pulmonary complaints in that building, with a lady almoner, not a trained nurse, but one who had experience of hospital work, who would have one or more visiting nurses working under her, to visit the homes of consumptive people. Thus the most desirable people would be kept in touch with, and they would be enlightened as to the best mode of life. He concluded by an appeal to help forward such a beneficent scheme. Last year the hospital was well supported with legacies, and less was spent on fuel and light, as the better and more economical "Osram" lamp had been used.

**Difficulties at Liverpool Eye Hospital.**

Peculiar interest was given to the recent annual meeting of the Liverpool Eye and Ear Hospital from the fact that the Lord Mayor, who presided, is vice-chairman of the Medical Inspection of Schools Sub-Committee, and the great financial difficulties of the hospital are credited by some people to the action of this committee in reporting to parents the results of their inspection. The general position at least is this : there have been about 12,000 new eye and ear cases, the hospital debt has risen to £3,246, and, as the Lord Mayor said, cases of disease must be reported to the parents, and those who can afford to pay must be prevented from sending their children to the hospitals. How many can afford to pay is not very easily ascertainable; a hospital in debt cannot afford to take them; a struggling practitioner cannot afford to be deprived of his fees for attending to them, and the education rate is already being grumbled at, so that the educational authorities will not very easily raise it again. This state of affairs is not only to be found in Liverpool, it is true, but the Eye and Ear Hospital is not supported there as well as similar intitutions where the same difficulties exist.

**Proposd Change at Luton Bute Hospital.**

At the recent annual meeting of the Luton Bute Hospital, Mr. Austin pointed out that, as the institution had long ceased to be a "Cottage Hospital," and the name of "Bute Hospital," had now no meaning for most people, it might be desirable to change its name to "Luton and South Beds. Hospital" or some similar title. He moved that the President, the Chairman, and the Hon. Secretary be asked to consider the matter before the enlarged building was opened. Dr. Doubble pointed out that so long as the institution was called "Cottage Hospital," people in the town and new firms would not expect to find a resident surgeon there. The annual report states the number of patients admitted to have been 366. It goes on to say that the works for reconstruction have now been started some three months, and it is anticipated that the buildings will be completed during the summer, when the hospital should fulfil all up-to-time-present requirements. On this re-construction scheme the Chairman said there was apparently a deficiency of something like £2,000. The work of re-construction had been forced upon them by the growth of the town, and also somewhat by the medical men telling them that the building which had hitherto been used for operations was not all that it should be, or all that was necessary, and as a matter of course the committee were now very anxious that the operating theatre should be furnished in the most up-to-date manner. The balance-sheet showed total receipts of £2,764, and this sum gave a surplus over the expenditure of £1,262, and £200 of this will be placed to the endowment fund.

**Brompton Hospital Annual Meeting.**

The Duke of Wellington presided at the annual Court of Governors of the Brompton Hospital for Consumption and Diseases of the Chest. Lord Cheylesmore pointed out that there was a deficit of some £6,000 on the year, the income being £31,713, and expenditure amounting to £37,651. The report stated that donations had been falling off of recent years, though there was a slight improvement on the financial condition of 1909, and letters are still being received from contributors to say that their subscriptions will be reduced on account of a diminished income. The number of in-patients admitted during the year was 1,686, and of new out-patients 6,258. An appeal is being issued for money with which to extend the clinical laboratories. The first donation of 100 guineas has come from Mr. F. W. Jackson.

**General Hospital, Tunbridge Wells.**

At the annual meeting of the Tunbridge Wells General Hospital on Wednesday last week, the Treasurer, Mr. F. Wadham Elers, M.A., J.P., presided over a large attendance. New in-patients numbered 680 during 1910, the out-patients reached 6,833, with 27,299 attendances. The income of the Hospital showed an increase under nearly all heads, but the ordinary income still falls short of the annual sum needed by some £500. Legacies, which were fortunately available, had to be drawn upon to this extent to meet the expenditure. The Hospital lost a most valued member of its staff by the death of Mr. W. H. Rix, who had been connected with the Institution as Hon. Surgeon and Consulting Surgeon for thirty-one years. The report met with the unanimous approval of the Governors, who showed their confidence in Mr. Elers by electing him Treasurer for the fourteenth consecutive year. The retiring members of the Committee were also re-elected.

**Birmingham General Hospital.**

The Lord Mayor of Birmingham, Alderman Bowater, took the chair in place of Viscount Newport, the president, at the annual meeting of the General Hospital. Among those present were Colonel Howard Wilkinson (chairman of the Board of Management), Sir John Holder, Bart., Sir Robert M. Simon, Mr. J. C. Vaudrey, Mr. George Heaton, and Mr. Howard J. Collins, house governor. The Lord Mayor said the in-patients were about the average number of the last few years, but he was sorry to see that out-patients now exceeded 67,000. Mr. Neville Chamberlain and others for some time past had been trying to induce the people of Birmingham not to run to them for trivial matters. Efforts had been made, and were still being made, though without such success as they hoped, for the provision of provident institutions in different parts of the city. The working classes contributed through the Hospital Saturday Fund the large amount of £10,000 a year to the hospitals, but while he recognised to the full the generosity of the working classes, he thought a very small amount regularly paid would be sufficient for them to have their ordinary ailments treated without their coming to overcrowd the out-patient departments of the hospitals. A penny a week for every man, woman, and child in the city, leaving a big margin for those too poor to give, would produce over £100,000 a year, and if they asked the doctors he thought they would say that would be a fair amount divided among them. He hoped they would realise therefore the great power in the penny. He proposed that the election of a president for the ensuing two years be left to the Board of Management. Sir John Holder, in seconding, said the increase in the nursing staff to 127, as compared with 106 when the new hospital was opened, arose from the enormous increase in the number of serious operations. With regard to the pressure on the out-patient department, they had done almost everything they could to reduce the number of applicants, and he hoped they would be successful, though last year there had been no reduction, but a large increase. The question of an enlarged institution would have to be considered, but at present the time was not ripe and the money was not available. The speaker further referred to the valuable work being done by the Jaffray Hospital in the relief of cases requiring prolonged treatment. Dr. Saundby said with regard to the idea of an enlarged hospital he hoped it would not be considered until they had obtained some much-needed improvements in the existing institution—a proper pathological department, and equipment at the Jaffray Hospital for open-air treatment. Mr. J. C. Vaudrey pointed out that the accumulated deficiency now amounted to £12,885. The Board was exceedingly grateful for the handsome legacy of £10,000 of the late Mr. John Feeney, which was paid by the trustees on the very day that it was due—five years after that gentleman's death. Mr. Neville Chamberlain, in proposing a vote of thanks to the medical and surgical staff, stated that in respect of the increase in the number of out-patients the attention that had been bestowed upon the matter had resulted in a more complete record of cases attended to.

**Royal Hospital for Diseases of the Chest.**

The Lord Mayor, who presided at the hospital, City Road, at the annual Court of Governors of the Royal Hospital for Diseases of the Chest, in moving the adoption of the report, said that the percentage of mortality was exceptionally low. Mr. Edward Gates seconded, and the report was adopted. The following are its principal facts and figures : During the year 762 patients were treated in the wards, and there were 25,540 attendances of patients in the out-patient department, the new out-patients numbering 6,034. No fewer than 226 patients were examined in the electrical department by the Röntgen rays. The total income for the year was £6,300, a decrease of £552, and the expenditure was £6,919, an increase of £333. It should not be forgotten that this was the first hospital to be established for the treatment of diseases of the chest.

**The Hospital's Decision at Coventry.**

Feeling that the town should be encouraged by the sight of active work on the part of the institution, the committee of the Coventry and Warwickshire Hospital has decided to begin part of that extension which it is hoped will form the city of Coventry's memorial to King Edward VII. This intention was announced only the other day at a meeting of the Memorial Fund Committee, when it was stated the funds already collected amounted to £7,388. In other words, lately the fund has been increasing at the rate of £1,000 a month, and, as Mr. Alfred Herbert remarked, if this rate can only be maintained neither the hospital nor the city will regret the decision to build. The pressure on the beds is a fact that is glaringly present to every member of the hospital committee every day of the week; the public only has to be persuaded of this fact to raise quickly the necesasry money for its abatement. The ward block, then, as the connecting link with the main building, will be proceeded with. Mr. E. M. Iliffe made the vigorous suggestion that the date of the laying of the foundation-stone should be promptly fixed, and a determined effort made to raise the money before then. That at any rate is the spirit in which the problem must be approached. In the meantime the local Coronation festivities will be used in the interests of the hospital, and a Godiva procession will almost certainly be held—a very popular amusement in Coventry, and one which has raised money for the hospital before.

**Loughborough Hospital and Dispensary.**

At the ninety-second annual meeting of the Governors, under the presidency of Mr. W. B. Paget, it was stated regretfully that the debt had unfortunately increased, chiefly owing to a falling off in workpeople's contributions. A new step is the purchase of an additional piece of land adjoining the hospital garden, which is used for convalescent patients, and since then advantage has been taken of an opportunity to acquire an additional piece of land kindly offered to the hospital. A ward for children has long been badly needed, and, thanks to a generous benefactor, the work of building is in hand. The President urged the necessity of support to the appeal now being issued for an endowment fund for the children's ward, the annual cost of maintaining which will be considerable.

